# A hidden Markov model to identify and adjust for selection bias: an example involving mixed migration strategies

**DOI:** 10.1002/ece3.1066

**Published:** 2014-04-19

**Authors:** John R Fieberg, Paul B Conn

**Affiliations:** 1 Department of Fisheries, Wildlife and Conservation Biology, University of MinnesotaSt. Paul, Minnesota, 55108; 2 National Marine Mammal Laboratory, Alaska Fisheries Science Center, NOAA National Marine Fisheries Service7600 Sand Point Way NE, Seattle, Washington, 98115

**Keywords:** Bayesian, facultative migrator, jags, latent variable, obligate migrator, partially observed state, state-space

## Abstract

An important assumption in observational studies is that sampled individuals are representative of some larger study population. Yet, this assumption is often unrealistic. Notable examples include online public-opinion polls, publication biases associated with statistically significant results, and in ecology, telemetry studies with significant habitat-induced probabilities of missed locations. This problem can be overcome by modeling selection probabilities simultaneously with other predictor–response relationships or by weighting observations by inverse selection probabilities. We illustrate the problem and a solution when modeling mixed migration strategies of northern white-tailed deer (*Odocoileus virginianus*). Captures occur on winter yards where deer migrate in response to changing environmental conditions. Yet, not all deer migrate in all years, and captures during mild years are more likely to target deer that migrate every year (i.e., obligate migrators). Characterizing deer as conditional or obligate migrators is also challenging unless deer are observed for many years and under a variety of winter conditions. We developed a hidden Markov model where the probability of capture depends on each individual's migration strategy (conditional versus obligate migrator), a partially latent variable that depends on winter severity in the year of capture. In a 15-year study, involving 168 white-tailed deer, the estimated probability of migrating for conditional migrators increased nonlinearly with an index of winter severity. We estimated a higher proportion of obligates in the study cohort than in the population, except during a span of 3 years surrounding back-to-back severe winters. These results support the hypothesis that selection biases occur as a result of capturing deer on winter yards, with the magnitude of bias depending on the severity of winter weather. Hidden Markov models offer an attractive framework for addressing selection biases due to their ability to incorporate latent variables and model direct and indirect links between state variables and capture probabilities.

## Introduction

An important assumption in observational studies is that sampled individuals are representative of some larger study population. Yet, this assumption is often unrealistic. When selection probabilities of observational units depend on the response of interest, sample and population summaries can be vastly different. Selection biases can also distort observed relationships between variables when selection probabilities depend on unmeasured characteristics related to both predictor and response variables. These problems are common when data are collected using voluntary surveys as individuals are more likely to respond if they hold strong opinions, often referred to as self-selection bias (White et al. 2005). Similarly, manuscripts are more likely to be submitted to academic journals and accepted for publication if they contain statistically significant results, leading to a well-known publication bias (Palmer 1999; Jennions and Møller 2002; Leimu and Koricheva 2004). Another notable example occurs when modeling species distributions using opportunistic locations (e.g., observations may tend to occur near roads or other areas frequently visited by observers; Kramer-Schadt et al. 2013) or habitat use if locations are frequently missed when animals are in heavy cover (Frair et al. 2010).

If the mechanisms leading to selection biases are known, or can be inferred from auxiliary data, then it is often possible to adjust for selection bias. For example, in telemetry studies, researchers often conduct stationary test trials where radiocollars are placed in a variety of habitats and fix rates are then estimated as a function of habitat features (Frair et al. 2010). These data can be used to model the probability of obtaining a successful fix as a function of covariates (environmental variables or time of day), and the fitted model can then be used to weight subsequent animal locations (by the inverse of the estimated fix success rate) when estimating home ranges or fitting habitat selection models (Horne et al. 2007; Frair et al. 2010). Similar methods have recently been suggested for correcting for self-selection biases in voluntary Web surveys when auxiliary data from a random sample of the target population are available to model selection probabilities in the voluntary survey (Schonlau et al. 2009).

Alternatively, one can attempt to model the selection process simultaneously with other important predictor–response relationships. This approach requires constructing the likelihood of the observed data, recognizing that the observed data likelihood is a function of both biological and observation (or selection) processes. As an example, Nielson et al. (2009) developed an approach to studying habitat selection that accounts for selection bias by simultaneously modeling habitat use and the probability of obtaining a successful fix. The probability of obtaining a successful fix depends on the (sometimes unobserved) habitat characteristics associated with the animal locations. The model is able to infer the characteristics of the unobserved locations from characteristics of "nearby" locations (in space and time) as well as from information on the distribution of distances moved between subsequent locations. Model-based solutions to the problem of selection biases will clearly be problem-specific and require considerable thought and creativity.

Our primary purpose of this study is to raise awareness of the potential for selection bias in ecological studies, but also to illustrate another interesting example where a model-based solution is possible. Specifically, we illustrate the problem of selection bias and a solution in the context of modeling mixed migration strategies of northern white-tailed deer (*Odocoileus virginianus*).

## Case Study: Modeling Mixed Migration Strategies of Northern White-tailed Deer

Many species migrate in response to seasonal changes in resource abundance or to escape predation, and decisions regarding if or when to migrate are often assumed to follow from environmental cues (Fryxell and Sinclair 1988; Nicholson et al. 1997; Fieberg and DelGiudice 2008; Meunier et al. 2008; Milner-Gulland and Fryxell 2011). Yet, in many populations, not all individuals migrate in all years. Much of our knowledge of mixed migration strategies comes from short-term studies. Inferring population-level characteristics from these studies is challenging because: (1) environmental variability is often limited; (2) individual migration strategies cannot be fully determined due to insufficient follow-up time; and (3) capture techniques may lead to selection biases, whereby the study cohort differs from the parent population targeted for inference. We describe how selection bias can be addressed by jointly modeling the population-level state distribution and the probability of first capture, and how a hidden Markov modeling framework can be used to account for uncertainty in group membership given a sequence of successive observations (i.e., migrate/do not migrate).

**Figure 1:**
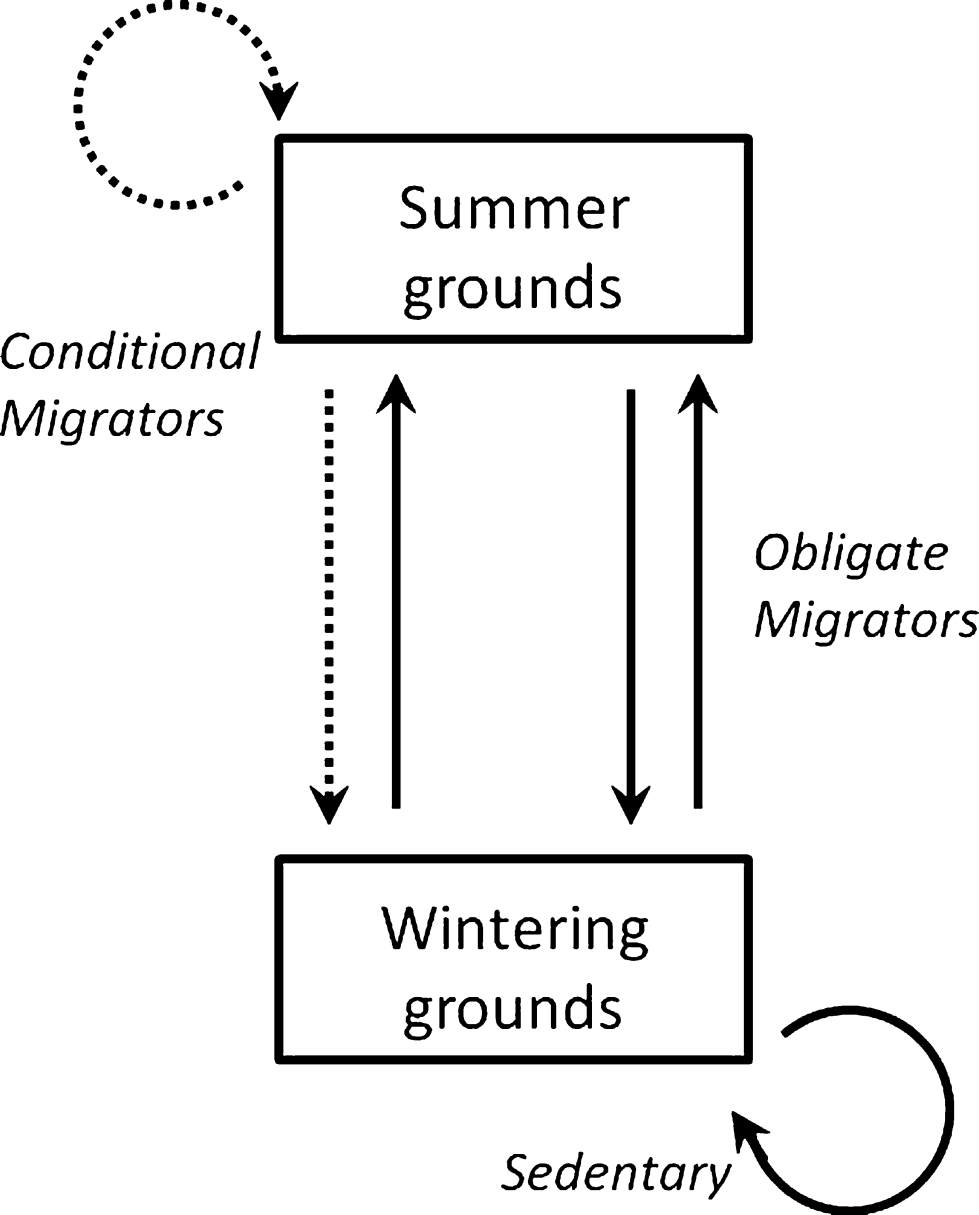
A depiction of three different migration strategies exhibited by individuals in the study cohort. *Sedentary* deer remain on wintering grounds all year and are not considered in our analysis. *Conditional* and *obligatory migrators* both return to summer grounds during the summer, but differ in wintering strategy. Obligate migrators (*z*_*i*_ = 1) make the trip to wintering grounds every year, while conditional migrators (*z*_*i*_ = 0) may or may not, depending upon winter severity. Solid arrows represent deterministic transitions, while dotted arrows represent probabilistic transitions.

Capture efforts associated with studies of northern populations of white-tailed deer (*Odocoileus virginianus*) have typically been concentrated on wintering areas, called "deer yards" (Nelson 1995; Van deelen et al. 1998; Fieberg et al. 2008). These areas are largely composed of dense conifer stands that serve as thermal cover and snow shelter for migratory deer as well as a few year-round residents (Fig. 1; DelGiudice et al. 2013). Deer captured on winter yards exhibit one of three different migration strategies. They may be as follows: (1) sedentary (i.e., year-round residents), with home ranges that overlap a winter yard; (2) obligate migrators that migrate between summer and winter grounds in every year; or (3) conditional migrators that migrate from summer grounds to winter grounds in a subset of years, usually in response to severe winter conditions, before (always) returning to summer grounds in the spring (Fig. 1).

Migration, as an evolutionary strategy, can be adaptive when resources or environmental conditions fluctuate seasonally in a predictable manner (Mueller and Fagan 2008). For white-tailed deer in northern climates, migration and winter yarding behavior can provide physiological, nutritional, and antipredatory benefits, due to reduced costs of movement during severe winters with deep snow (Taylor and Taylor 1977; Messier and Barrette 1985; Nelson and Mech 1986; Fryxell and Sinclair 1988; Fieberg et al. 2008). In a 15 year study of adult (> 1.0 year old) female deer, Fieberg et al. (2008) found the proportion of deer migrating from spring–summer–autumn range (hereafter "summer grounds") to winter grounds varied nonlinearly with an index of winter severity. These findings are significant ecologically, but they also have important consequences for the analysis and interpretation of these data. For example, commonly applied naïve migration classification schemes, whereby individuals are considered obligate migrators if they are observed to migrate in every year they are followed, will be influenced not only by study duration but also by the severity of the winters encountered during the study. Fieberg et al. (2008) found the probability of naively classifying a deer as an obligate migrator was inversely proportional to the number of years each deer was followed (range 2–7), and was also lower for those deer observed during one or more mild winters. Because deer were captured on winter yards, the pattern of winter severities was also thought to influence the composition of the study cohort. Specifically, more obligate migrators were thought to be captured during mild winters. Similar concerns have been raised by others studying migration patterns of deer in northern climates (see e.g., Van deelen et al. 1998).

Fieberg et al. (2008) used a deterministic model to illustrate how selection biases might influence the composition of the study cohort, and subsequently, the proportion of deer observed to migrate in future years. In years with mild winters, the proportion of deer that migrated was highly dependent on the proportion of obligate migrators in the study cohort. By contrast, the proportion of deer migrating during severe winters was always > 90%, regardless of the composition of the study cohort. Similar patterns were evident in their empirical data; the proportion of deer migrating during mild winters was more variable than during severe winters, and Fieberg et al. (2008) attributed these results to annual variation in the composition of the study cohort.

Investigation of empirical data and application of deterministic modeling have been useful tools for detecting selection biases associated with radio collaring efforts, but are insufficient to permit unbiased estimation of population-level migration parameters. This is unfortunate, as the composition and migratory disposition of the study cohort will give an unclear picture of behavior of the population as a whole. To address these concerns, we developed a Bayesian model that incorporates a vector of partially observed states reflecting each individual's migration strategy (conditional vs. obligate migrator). Using this model, we reanalyze the data from Fieberg et al. (2008). Our specific objectives are to: (1) provide a robust estimate of the proportion of obligate migrators in the population; (2) quantify the impact of selection biases on estimates of the proportion of deer migrating in each year; and (3) more clearly identify the effect of winter severity on migration patterns. More generally, we hope to highlight the potential for selection biases in ecological studies and demonstrate how one can estimate and adjust for these biases using hidden Markov models.

## Materials and Methods

### Data

During January–March 1991–2006, female deer ≥ 0.5 years old were captured on wintering areas within a 791 km^2^ study area in northern Minnesota, USA. Inclusion of summer grounds expanded the study area to 1,865 km^2^ (Powell et al. 2005). Deer were captured primarily by Clover traps (Clover 1956), but rocket-nets and net-gunning were used for a small percentage (<5% each) of the total capture (DelGiudice et al. 2005). Deer were fitted with very high frequency (VHF; Telonics, Mesa, Arizona; Advanced Telemetry Systems, Isanti, Minnesota) or global positioning system (GPS; Advanced Telemetry Systems) radiocollars. Animal capture and handling protocols were approved by the University of Minnesota's Institutional Animal Care and Use Committee and are described in further detail elsewhere (DelGiudice et al. 2005).

Deer with VHF collars were monitored for survival from fixed-wing aircraft 1–3 times per week (DelGiudice et al. 2006) and located for habitat analyses less frequently (DelGiudice et al. 2013). Deer with GPS collars were monitored daily, with locations attempted every 1–4 h. Deer were followed until they died or their collared failed, and new individuals were recruited into the study annually to replace these individuals (Table 1). Movements of ≥2 km were considered to be migratory when summer and winter grounds did not overlap. Roughly, 1/3 of the deer captured were sedentary (i.e., nonmigratory). These deer are easily identified because they remain on wintering grounds all year long, whereas conditional and obligate migrators always migrate to summer grounds in the spring (Fig. 1).

**Table 1:** Winter severity, total number of radiocollared migratory white-tailed deer (i.e., cohort size) followed in each year, number of migratory deer newly recruited into the study cohort, and proportion of the study cohort migrating during winters 1991–1992 to 2005–2006

Winter	WSI^1^	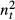	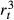	Proportion migrating ( 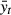 )
1991–1992	86	11	11	0.73
1992–1993	124	16	13	1.00
1993–1994	126	30	16	0.97
1994–1995	61	31	14	0.48
1995–1996	195	21	4	0.81
1996–1997	159	31	23	0.97
1997–1998	50	35	19	0.14
1998–1999	46	17	1	0.35
1999–2000	45	22	12	0.27
2000–2001	153	16	1	0.63
2001–2002	45	31	24	0.61
2002–2003	58	32	9	0.38
2003–2004	42	24	2	0.92
2004–2005	108	17	6	0.94
2005–2006	45	23	13	0.35

1 WSI is calculated as the maximum cumulative number of days with temperatures ≤17.7^∘^+ the cumulative number of days with snow depths ≥38 cm.

^2^Number of migratory deer monitored in year *t*.

^3^Number of radiocollared animals newly recruited into the study cohort during year *t*.

Minimum and maximum ambient temperatures and snow depths were recorded daily at designated nonforested (i.e., open) locations in the study area during January–March 1991–2005 (DelGiudice 1998; DelGiudice et al. 2006). Data for November–December and late March–May 1990–2005, and November 2005–April 2006 were obtained from a weather station at Grand Rapids, Minnesota. A winter severity index was calculated by accumulating one point for each day with an ambient temperature ≤17.7^∘^C (temperature-day) and one point for each day when snow depth was ≥38 cm (snow-day) during 1 November–31 April. Maximum winter severity indices (hereafter WSI) in each winter ranged from 42 to 195 during the course of the study.

### Model formulation

Fundamentally, the initial probability of capturing and radiocollaring animals subscribing to different migration strategies is composed of two components: the proportion of animals (without collars) in the population belonging to each migratory group *s* at time *t*, *π*_*s*,*t*_, and the (possibly time specific) probability an animal is captured given that it is a member of group *s*, *p*_*s*,*t*_. Using Bayes rule, the probability that a randomly captured deer in year *t* will have migration strategy (*S* = *s*) is given by:


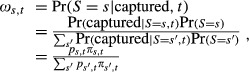
(1)

where the summation is taken over all possible migratory strategies. In some cases, we might wish to further expand the model for *p*_*s*,*t*_; for instance, we might want to model *p*_*s*,*t*_ as the product of the probability that an animal is available for capture (i.e., whether or not it is on a winter yard), *θ*_*s*,*t*_, and the probability of capture (conditional on it being available), *p*_*t*_. The subscripts in this case indicate that migration strategies influence the probability that an animal is available for capture, but not the probability of capture (once available). Under this formulation, Eq. 1 becomes


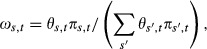
(2)

with the probability of detection, *p*_*t*_, canceling out. Our strategy will be to employ Eq. 2 when modeling the state distribution associated with deer captured on winter grounds. As with previous studies, a primary goal of our work was to quantify the role of winter severity in determining whether or not deer migrate to winter yards. Thus, we further simplify the problem by focusing only on the population of obligate and conditional migrators, excluding year-round residents (i.e., sedentary deer that have home ranges overlaping wintering areas). Conditional and obligate migrators always migrate to summer grounds in the spring, making it easy to identify year-round residents by the lack of a spring migration (Fig. 1). Because home ranges of sedentary animals can expand and contract (and many sedentary animals may never be exposed to sampling), including these animals in the analysis had the potential to obscure rather than clarify the impact of sampling bias on annual composition estimates.

While obligates migrate every year, conditional migrators are more likely to migrate during severe winters. To capture this dynamic, we modeled the logit probability of migration in year *t* for conditional migrators as a linear function of the WSI in year *t*, *x*_*t*_. Specifically, let *y*_*i*,*t*_ = 1 if the *i*^*th*^ deer migrates in year *t* and 0 otherwise, and *z*_*i*_ = 1 if the *i*^*th*^ deer is an obligate migrator and 0 otherwise (*i* = 1, 2,…,*n*). Then:


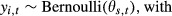



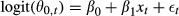



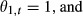



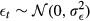


Or, unconditionally:


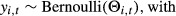



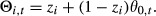


Here, 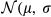
^2^) denotes a normal (Gaussian) distribution with mean *μ* and variance *σ*^2^. The migration strategy indicators, *z*_*i*_(*i* = 1,2,…,*n*), are only partially observed. Deer that do not migrate from their summer grounds in ≥1 year are known to be conditional migrators (i.e., *z*_*i*_ = 0), but the converse is not necessarily true. Thus, *z*_*i*_ is treated as a latent (unknown) parameter for deer that migrated in all years that they were observed.

To account for the selection bias that occurs from capturing deer on winter yards, we applied Eq. 2. Specifically, we modeled


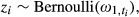


where *t*_*i*_ indicates the sampling occasion on which individual *i* was initially captured and collared. In our special case of only two migratory classes (conditional and obligatory migrants), the expression for *ω*_1,*t*_ (Eq. 2) simplifies to


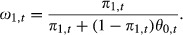
(3)

Thus, the probability that a captured deer will be an obligate migrator will be higher in mild years (i.e., when *θ*_0,*t*_ is small).

Population-level state distributions, *π*_*s*,*t*_, may vary over time owing to (1) expansion and contraction of the relative abundance of each migratory sector; and (2) selection bias in the marking (i.e., capture and collaring) process drawing down the number of unmarked animals in each sector at different rates. Although we did not consider the latter to be important in our deer example because of the relatively small proportion of the population that is caught and radiocollared each year (Table 1), this factor may be important to consider in other applications. We assumed *π*_1,*t*_ varied smoothly over time, and modeled changes in logit(*π*_1,*t*_) using natural cubic regression splines with 2 degrees of freedom:


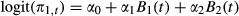
(4)

We created the values of the spline basis functions (i.e., *B*_1_ and *B*_2_) using the "ns" function in the splines library of Program R (Chambers and Hastie 1993; R Development Core Team 2011), placing a single interior knot at year 5, corresponding to the most severe winter during the study.

Rather than determine the marginal likelihood for each *y*_*i*,*t*_ by integrating over the latent variables (*ε*_*t*_, *z*_*i*_), we used Markov chain Monte Carlo (MCMC) to numerically integrate over the latent variables using a Bayesian formulation of the problem. We specified 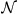
(0, 3) priors for *β*_0_ and *α*_0_ because reverse transformation results in an approximately uniform distribution on (0,1). We specified 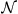
(0, 10) priors for *β*_1_, *α*_1_, *α*_2_. Lastly, we specified a uniform(0, 10) prior for *σ*_*ε*_. We used the R2jags package in Program R to facilitate estimation with jags (R Development Core Team 2011; Su and Yajima 2012) and assessed convergence by inspecting trace plots and Gelman–Rubin statistics (Brooks and Gelman 1998). After convergence, we generated an additional 90,000 samples (30,000 from each of three chains) from the posterior distribution of each parameter and 90,000 values from the posterior predictive distribution of each *z*_*i*_.

To investigate the potential selection bias in each year of the study, we compared the estimated proportion of obligate migrators in the population (of animals without collars), 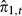, to an estimate of the proportion of deer in the study cohort (animals with collars) that were obligate migrators, 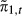, defined as follows:


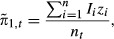
(5)

where *n*_*t*_ is the number of radiocollared individuals in year *t*, *n* is the total number of migratory animals followed throughout the survey, and *I*_*i*_ is an indicator taking on a value of 1 if individual *i* is in the study in year *t* and 0 otherwise. Lastly, we calculated the expected value of *z*_*i*_ for each deer in the study, as the average of the *z*_*i*_'s across all MCMC iterations.

## Results

The mean annual cohort size was 24 and ranged from a low of 11 in the first year of the study to a high of 35 during the winter of 1996–1997 (Table 1). Overall, 168 deer were monitored for at least one winter migration period. The proportion of the study cohort that migrated ranged from a low of 0.14 in the winter of 1997–1998 (WSI = 46) to 1.0 in 1992–1993 (WSI = 124) (Table 1).

**Figure 2:**
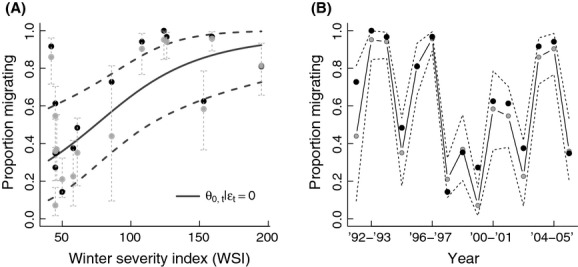
Model-based estimates of the probability of migrating: (A) as a function of the Minnesota Department of Natural Resource's winter severity index (WSI) and (B) as a function of time. Black points in both panels give the proportion of the study cohort migrating in each year. Gray circles depict model-based estimates of the proportion of deer expected to migrate in each year = *π*_1,*t*_ + (1−*π*_1,*t*_)*θ*_0,*t*_. In panel (A), the black solid line depicts the probability of migrating for conditional migrators (*θ*_0,*t*_|*ε*_*t*_ = 0). In all cases, dotted lines indicate 90% Bayesian credible intervals.

**Figure 3:**
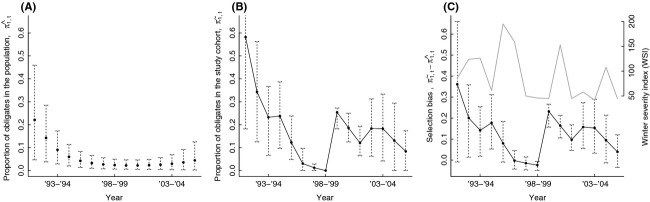
Selection bias associated with winter capture on deer yards: (A) estimated proportion of obligate migrators in the unmarked population, 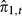; (B) estimated proportion of obligate migrators in the study cohort, 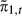; (C) Selection bias quantified as the difference in the proportion of obligate migrators in the study cohort and unmarked population, 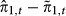. The gray line in Panel C (right axis) depicts the winter severity index (WSI) in each year. Points represent posterior means, and dotted lines represent Bayesian 90% credibility intervals.

The estimated probability of migrating for conditional migrators increased with WSI and varied considerably over the course of the study (Fig. 2A and B). Estimates of the proportion of obligate migrators in the (un-collared) population, 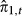, ranged from a high of 0.22 (90% Bayesian credible interval = 0.05,0.46) during the first year of the study to a low of 0.02 (90% Bayesian credible interval = 0.005, 0.05) during the latter part of the study (Fig. 3A). Estimates of the proportion of obligates in the *study cohort*, 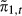, were consistently higher than 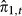 (Fig. 3B), except during a span of 3 years surrounding back-to-back severe winters.

**Figure 4:**
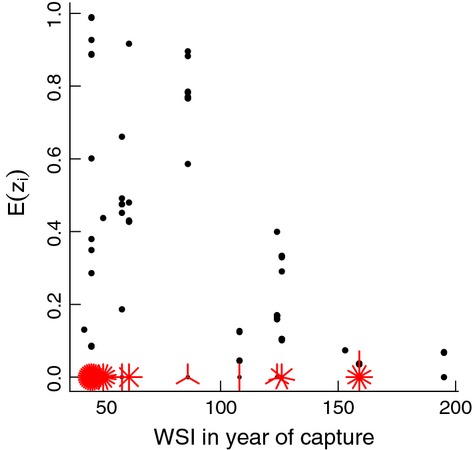
Likelihood of being an obligate migrator as a function of winter severity in the year of capture. For each deer (*i*), *E*[*z*_*i*_] represents the probability the individual is an obligate migrator. Deer that do not migrate in at least 1 year are known to be conditional migrators (*z*_*i*_ = 0) (multiple observations with *z*_*i*_ = 0 are represented by "petals" in the sunflower plot). For all other deer, *z*_*i*_ is a latent variable, with a different value sampled during each MCMC iteration. Values of E[*z*_*i*_] shown here are averages across 90,000 MCMC iterations.

We can gain further insights into the potential for selection bias by comparing estimates of the proportion of obligate migrators in the study cohort (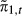) to that of the un-collared population (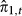) over time. Initial estimates of 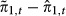 suggest that obligate migrators were over-represented in the study cohort (Fig. 3C). Selection biases were reduced, however, by the back-to-back severe winters in 1995–1996 and 1996–1997 (WSI = 195 and 159, respectively), which helped to recruit more conditional migrators into the study population. These two severe winters were then followed by a series of 3 unprecedentedly mild winters (WSI = 50, 46, and 45; Table 1), which caused the study population to revert back to one in which obligate migrators were once again over-represented (Fig. 3C). Although conditional migrators (*z*_*i*_ = 0) were captured in all years, those deer identified as most likely being obligate migrators (i.e., those with E(*z*_*i*_) close to 1) were most often captured and collared during mild winters (Fig. 4). Overall, these results support our hypothesis that selection biases occur as a result of capturing deer on winter yards and that the magnitude of the bias depends on the severity of winter weather.

## Discussion

### Mixed migration studies and hidden Markov models

Several studies have highlighted the importance of winter weather in determining whether and when deer in the northern part of their range migrate (Nelson 1995; Sabine et al. 2002; Ramanzin et al. 2007; Fieberg et al. 2008). Deer in these studies have typically been captured on winter yards where deer congregate in high numbers, and thus, capture efficiencies are greatest (Barrett et al. 2008). Because conditional migrators are less likely to be present on winter yards during mild winters, Nelson (1995) and Fieberg et al. (2008) questioned whether selection biases might influence estimates of important population parameters. Further, most studies tend to be short term (3–5 years), with limited follow-up time for individual animals (1–2 years). As such, it can be difficult to characterize individual deer as obligate or conditional migrators or to accurately estimate the proportion of deer in the population exhibiting each of these strategies. We developed a Bayesian approach using partially observed latent variables and utilized long-term data, to help overcome both of these challenges (selection bias and limited follow-up).

A recent focus in the analysis of marked animals has been to develop models that explicitly allow for imperfect observations of the "state" of an animal. For instance, Pradel (2005) proposed an approach for analyzing multistate mark–recapture data where the ecological state of interest (e.g., breeder/nonbreeder) is potentially decoupled from the observation type (e.g., observed near nest, observed on nest, observed away from nest). This type of hidden Markov model (Zucchini and MacDonald 2009), also coined a "multievent" model in the context of mark–recapture estimation, has been used by numerous authors to study topics as diverse as disease ecology (e.g., Conn and Cooch 2009; Lachish et al. 2011), breeding ecology (e.g., Lescroël et al. 2009), and animal migration or dispersal (Péron et al. 2010; Sanz-Aguilar et al. 2012; Lok et al. 2013). When observations are probabilistically related to underlying states, multievent models allow estimation of state-specific survival and transition probabilities. However, the canonical formulation for multievent models does not provide any linkage between the distribution of states obtained at initial capture and the distribution of states in the population (Kendall et al. 2012). As such, naïve interpretation of initial state distributions from multievent models as population-level proportions of animals belonging to each state (e.g., migratory group) can be seriously compromised anytime there is selection bias in initial marking.

In certain cases, it is possible to make inferences about population-level state distributions using data from marked animals. For instance, Thorup and Conn (2009) combined a finite mixture distribution with a multistate mark–recovery model to estimate proportions of sub-Saharan seasonal bird migrants. However, their approach assumed that investigators were equally likely to mark birds on summer grounds regardless of migratory type (and thus cannot cope with selection bias). Kendall et al. (2012) showed that integrating capture–recapture data into a hidden Markov modeling framework allowed unbiased estimation of state distributions. In their case, a population closure assumption allowed estimation of the state-dependent probabilities of first capture, which in turn permitted estimation of population-level state distributions.

Our approach built on hidden Markov models, which allowed us to separate underlying migration states (e.g., conditional or obligate migrant) from observation type (migrate/did not migrate). However, unlike most hidden Markov models for mark–recapture–recovery data, our model provides a framework for estimating the proportion of obligate migrators in the unmarked population, *π*_1,*t*_. This value is synonymous with population-level stage structure in year 1 of the study and will likely be a good approximation in later years, especially for populations where the marking process does not appreciably alter the stage structure of the unmarked population. Interestingly, the estimates of *π*_1,*t*_ (Fig. 3A) are in line with the estimated probability of naively classifying a deer as an obligate migrator, conditional on the deer being followed for ≥7 years and experiencing a minimum WSI of 51 (see Fig. 1, left panel from Fieberg et al. 2008). The latter was estimated by fitting a logistic regression model to naive deer classifications (*z*_*i*_ = 1 for deer observed to migrate in all years they are followed and 0 otherwise) as a function of an individual's follow-up time and the minimum WSI experienced while under observation.

In developing our model, we assumed conditional migrators could not be captured unless they migrated to winter grounds. This assumption was reasonable for these data, as all of the capture efforts were concentrated on winter yards and capture efforts did not begin until late in the winter after deer had migrated. Nonetheless, this assumption may not be realistic if a wider distribution of capture effort (e.g., on and off deeryards) is employed. This key assumption could be relaxed by using a more general model for the selection bias. For example, one could assume: logit[*ω*_1,*t*_] = *γ*_0_+*γ*_1_*x*_*i*,*c*_, where *x*_*i*,*c*_ is the WSI during the year individual *i* was captured, and *γ*_0_ and *γ*_1_ are additional regression parameters to be estimated. Although this modification would allow one to model and adjust for selection biases, the advantage of the current formulation is that it provides a direct estimate of *π*_*s*,*t*_.

In our application, we made the simplifying assumption that the population was composed of a mixture of two types of animals: those that always migrate (obligates) and others that migrate in response to winter weather (conditional migrators). Nonetheless, it would be easy to extend the approach to populations exhibiting a variety of partial migration strategies (Mueller and Fagan 2008; Cagnacci et al. 2011). Further, random effects could be used to allow for a more continuous characterization of migration propensity. For example, one could define *z*_*i*_ to be a normally distributed latent variable, capturing the propensity of individual *i* to migrate. The probability of migrating could then be specified using: logit[*Θ*_*i*,*t*_]=*β*_0_+*β*_1_*x*_*t*_+*z*_*i*_. Selection biases could once again be accounted for by allowing *z*_*i*_ to depend on the winter severity during the year of capture.

Our primary focus in this study was on estimating migration-related parameters, as opposed to other life-history parameters like survival. As such, we were able to greatly simplify model construction by conditioning our model on animals that were known to be alive. As with hidden Markov models for mark–recapture–recovery data (cf. Pradel 2005), it should be possible to extend the model we have developed here to include state-specific survival parameters (i.e., with different survival parameters that depend on migration strategy). Such an extension would be useful for addressing ecological and evolutionary tradeoffs associated with different migration strategies and will be explored in future work.

### Importance of selection biases in ecological studies

Selection biases are likely to be prevalent in many ecological studies, and in such cases, researchers should make attempts to study and correct for these biases. One area that has received much attention, particularly lately, is the potential bias that can result from missed locations in animal telemetry studies (e.g., Frair et al. 2010; Conn et al. 2012). As discussed in the introduction, inverse weighting and model-based solutions to selection bias have both been suggested in this context. Researchers should also consider the potential for selection biases when recruiting individuals into these studies. In addition to selection biases arising from the location of traps, certain capture techniques may select for individuals that tend to be in poorer condition. For example, baited traps may select for individuals that have trouble competing for food resources. By using multiple trapping methods (e.g., clover traps with baits and net guns), it may be possible to test this hypothesis. Similarly, it may be possible to adjust for potential selection biases (e.g., in survival analyses) by modeling individual condition as a function of trap type. The ability to incorporate latent states and also model both direct and indirect links between variables make Bayesian methods an attractive framework for addressing these important issues.

## Data Accessibility

We used the R package, knitr (Xie 2013), to produce html files with all computer code and output, which we supply as Supporting Information. Data from this paper have been deposited in the Dryad repository (Fieberg 2014).

## Supporting Information

**Appendix S1.** R and Jags code used to fit the hidden Markov model.

**Appendix S2.** R code used to produce all plots in the paper.
