# Supplementary material for: A hidden Markov model to identify and adjust for selection bias: an example involving mixed migration strategies
Source: Ecol Evol. 2014 Apr 19;4(10):1903–12. doi: 10.1002/ece3.1066 (PMC4063483; doi:10.1002/ece3.1066)
Supplement: Supplementary file 1 [file ece30004-1903-SD1.pdf]

Bayesian Migration Paper

- PAPER: Using telemetry and a Bayesian hidden Markov model to identify mixed migration strategies while accounting for sample selection bias
- PROGRAM: BayesFit_ns.R
- DESCRIPTION: JAGS model for deer migration
- PURPOSE: Fits Bayesian model, output MCMC iterates for plotting
- AUTHOR: John Fieberg

Make sure everything points to the correct working directory (will work when cutting and pasting code or "spinning" using the 'spin' function in the knitr package)

```
wd <- ifelse(basename(getwd()) == "Scripts", gsub("/Scripts", "", getwd()),
    getwd())
opts_knit$set(root.dir = wd)
```

remove anything in the working memory

```
rm(list = ls(all = TRUE))
```

Get a random seed for random number generation & save it so we can reproduce the results.

```
exists.seed <- try(load("./data/rseed.Rdata"))
if (class(exists.seed) == "try-error") {
    set.seed(floor(runif(1, 0, 1e+05)))
    seed.save = .Random.seed
    save(seed.save, file = "./data/rseed.Rdata")
}
set.seed(seed.save)
```

load libraries

```
library(R2jags)
library(doBy)
library(R2WinBUGS)
library(splines)
```

Read in migration data for migrators

```
fmig <- read.csv("./data/migdat.csv")
```

Number of unique deer of each type and total

```
nindiv <- length(unique(fmig$deer.id))
```

Total number of obs (deer-years)

```
nobs1 <- nrow(fmig)
```

number of years in the study

```
nyrs <- length(unique(fmig$fall))
```

Reorder years and capture years so they start from 1

```
year <- fmig$fall - min(fmig$fall) + 1
cap.year <- fmig$capyear - min(fmig$fall) + 1
```

Get unique capture year wsi for each deer, and subtract mean to center

```
temp <- summaryBy(cwsi ~ deer.id, FUN = min, data = fmig)  #(note, min = max = unique value per
deer)
cwsi2 <- temp[, 2]
cwsi <- (cwsi2 - mean(cwsi2))
```

Get unique capture years for each deer

```
capyears <- tapply(cap.year, fmig$deer.id, max)  # note max=min=mean,etc here
deer.id <- fmig$deer.id
```

migration indicator

```
y <- fmig$cens  # equal 1 if migrated, 0 if did not
```

Wsi values from: Fieberg, J., D. W. Kuehn, and G. D. DelGiudice. 2008. Understanding variations in autumn migration of northern white-tailed deer by long-term study. Journal of Mammalogy 89:1529-1539.

```
wsis2 <- c(86, 124, 126, 61, 195, 159, 50, 46, 45, 153, 45, 58, 42, 108, 45)
wsis <- (wsis2 - mean(wsis2))
```

Create partially observed indicator variable of the migration strategy, z:

- z = 0 for conditional migrators
- z = 1 for obligate migrators

Identifying z:

1. If the deer migrates from winter to summer range, and does NOT migrate back to winter range in at least 1 year, then we know it is a conditional migrator (z=0)
2. If the deer migrates in every year it is followed, then we cannot say for sure it is an obligate migrator.

```
z <- rep(NA, nindiv)
inds <- tapply(1 - fmig$cens, deer.id, max)  # = 1 if did not migrate in at least 1 year
z[inds == 1] <- 0  # we know these deer are NOT obligate migrators
```

State probabilities for the unmarked segment of the population

Use regression splines with 2 degrees of freedom to model changes in the poportion of (uncollared) obligate migrators (pi[1,t]), with pi[0,t]= 1-pi[1,t]. Create spline basis (with knot at year = 5 = 1995-1996 the most severe winter of the study) for modeling pi[1,t]

```
ns.yr <- matrix(as.numeric(ns(1:15, knots = 5)), 15, 2)

# Bayesian model for JAGS
```

```
# note: ns.yr[,1] = B1 and ns.yr[,2] = B2

model_mig <- function() {
    # Probability migrate | conditional migrator (depends on wsis)

    # Priors for regression parameters associated with conditional migrators
    bo ~ dnorm(0, 0.333)
    b1 ~ dnorm(0, 0.1)

    # Priors for random effects to allow for overdispersion
    sigma_epsilon ~ dunif(0, 10)
    taup <- 1/(sigma_epsilon * sigma_epsilon)
    for (k in 1:nyrs) {
```

```
        eps[k] ~ dnorm(0, taup)
        logit.theta[k] <- bo + b1 * wsis[k] + eps[k]
        theta[k] <- exp(logit.theta[k])/(1 + exp(logit.theta[k]))
    }


    # pi[1,t] and pi[2,t] vary with year, with non-linear effect modeled using
    # ns(year,knots=5)...  I.e., a 2-df regression spline...below are the priors
    # for the regression parameters for pi[1,t] and pi[2,t]
    alpha_0 ~ dnorm(0, 0.333)
    alpha_1 ~ dnorm(0, 0.1)
    alpha_2 ~ dnorm(0, 0.1)


    # Below, pi.1 = P(obligate migrator & not previously captured)
    for (k in 1:nyrs) {
        logit.pi1[k] <- alpha_0 + alpha_1 * ns.yr[k, 1] + alpha_2 * ns.yr[k,
            2]
        pi.1[k] <- exp(logit.pi1[k])/(1 + exp(logit.pi1[k]))
    }
    # Below, w = P(obligate migrator | captured in year t)
    for (i in 1:nindiv) {
        w.1[i] <- pi.1[capyears[i]]/(pi.1[capyears[i]] + (1 - pi.1[capyears[i]]) *
            theta[capyears[i]])
        z[i] ~ dbin(w.1[i], 1)
    }
    # Below, Theta = probability deer migrates (=1 if obligate, depends on wsi
    # if not)
    for (i in 1:nobs1) {
        Theta[i] <- ifelse(z[deer.id[i]] == 1, 1, theta[year[i]])
        y[i] ~ dbin(Theta[i], 1)
    }
}

### Base model
write.model(model_mig, "./output/modelmigrate_ns.txt")

# Initial values
init.val <- function() {
    w.1 <- rep(0, nindiv)
    bo <- 0
    b1 <- 0
    alpha_0 <- rnorm(0, 1/0.333)
    alpha_1 <- dnorm(0, 1/0.1)
    alpha_2 <- dnorm(0, 1/0.1)
    list(bo = bo, b1 = b1, alpha_0 = alpha_0, alpha_1 = alpha_1, alpha_2 = alpha_2)
}

set.seed(19279)
jagsfit.mig <- jags(data = c("z", "wsis", "capyears", "deer.id", "nyrs", "nindiv",
    "year", "y", "nobs1", "ns.yr"), parameters.to.save = c("bo", "b1", "alpha_0",
    "alpha_1", "alpha_2", "pi.1", "theta", "z"), working.directory = "./output",
    inits = init.val, n.iter = 40000, n.burnin = 10000, model.file = model_mig,
    n.thin = 1, progress.bar = "none")
```

```
## Compiling model graph
##    Resolving undeclared variables
##    Allocating nodes
##    Graph Size: 1926
##
## Initializing model
```

Test for convergence (using Rhat)

```
jagsfit.mcmc <- as.mcmc(jagsfit.mig)
```

Parameters of interest for Gelman stat, etc

```
parnames <- c("bo", "b1", "alpha_0", "alpha_1", "alpha_2", "theta")
```

Convergence: looks good

```
gelman.diag(jagsfit.mcmc[][, colnames(jagsfit.mcmc[[3]]) %in% parnames])
```

```
## Potential scale reduction factors:
##
##         Point est. Upper C.I.
## alpha_0          1       1.00
## alpha_1          1       1.00
## alpha_2          1       1.00
## b1               1       1.01
## bo               1       1.00
##
## Multivariate psrf
##
## 1
```

Summaries

```
summary(jagsfit.mcmc[][, colnames(jagsfit.mcmc[[3]]) %in% parnames])
```

```
##
## Iterations = 1:30000
## Thinning interval = 1
## Number of chains = 3
## Sample size per chain = 30000
##
## 1. Empirical mean and standard deviation for each variable,
##    plus standard error of the mean:
##
##            Mean    SD Naive SE Time-series SE
## alpha_0 -1.4500 0.870 2.90e-03       0.017761
## alpha_1 -4.4325 1.921 6.40e-03       0.037533
## alpha_2 -1.0085 1.378 4.59e-03       0.013180
## b1       0.0263 0.012 3.99e-05       0.000286
## bo       0.3934 0.555 1.85e-03       0.012495
##
## 2. Quantiles for each variable:
##
##            2.5%     25%     50%     75%   97.5%
## alpha_0 -3.4074 -1.9647 -1.3681 -0.8487  0.0425
## alpha_1 -8.1263 -5.7131 -4.4749 -3.2018 -0.4751
## alpha_2 -4.2716 -1.7548 -0.8506 -0.0819  1.2769
## b1       0.0038  0.0187  0.0259  0.0335  0.0512
## bo      -0.7225  0.0437  0.3929  0.7460  1.5057
```

Save everything created from this program. First, however, create an object holding today's date.

```
today.date <- date()
save.image(file = "./output/Modelfit_ns.Rdata")
```
