# Supplementary material for: A hidden Markov model to identify and adjust for selection bias: an example involving mixed migration strategies
Source: Ecol Evol. 2014 Apr 19;4(10):1903–12. doi: 10.1002/ece3.1066 (PMC4063483; doi:10.1002/ece3.1066)
Supplement: Supplementary file 2 [file ece30004-1903-SD2.pdf]

Bayesian Migration Paper

- PAPER: Using telemetry and a Bayesian hidden Markov model to identify mixed migration strategies while accounting for sample selection bias
- PROGRAM: Plotmig_ns.R
- DESCRIPTION: Reads in MCMC iterates from BayesFit.R and creates all plots contained in the paper.
- AUTHOR: John Fieberg

Make sure everything points to the correct working directory (will work when cutting and pasting code or “spinning” using the 'spin' function in the knitr package)

```
spin <- ifelse(basename(getwd()) == "Scripts", 1, 0)
wd <- ifelse(basename(getwd()) == "Scripts", gsub("/Scripts", "", getwd()),
    getwd())
opts_knit$set(root.dir = wd)
```

remove anything in the working memory

```
rm(list = ls(all = TRUE))
```

Load libraries

```
library(gplots)
library(doBy)
```

Now, load in most recent output from BayesFit_ns.R

```
load("./output/Modelfit_ns.Rdata")
```

Determine the number of new deer collared in each year

```
n.cap <- summaryBy(fall ~ deer.id, FUN = c(min), data = fmig)
table(n.cap$fall.min)
```

```
##
## 1991 1992 1993 1994 1995 1996 1997 1998 1999 2000 2001 2002 2003 2004 2005
##   11   13   16   14    4   23   19    1   12    1   24    9    2    6   13
```

Determine the number of deer Pull off some basic information

```
n <- length(jagsfit.mig$BU$sims.list$bo)  # number of MCMC iterates
zs <- jagsfit.mig$BU$sims.list$z  # latent indicator variables for each deer x each iterate


# # Code to produce quantities included in the Figure 2 and 3
```

Set up matrices to hold yearly estimates (and upper, lower CI limits)

```
wsi.yr <- wsi.yrc <- matrix(NA, nyrs, 1)  # winter severity measures
pi1.tilde.d <- pi1.d <- matrix(NA, nyrs, 3)  # Proportion of obligates (study cohort of radiocollared animals,
 non-radiocollared animals in the population)
diffpop.samp <- matrix(NA, nyrs, 3)  # Differences in pi1.tilde and pi.1^
Thetap <- matrix(NA, nyrs, 3)  # Marginal probability of migrating for deer that are not radiocollared
p_mig <- matrix(NA, nyrs, 1)  # Proportion of the study cohort migrating in each year
for (i in 1:nyrs) {
    # loop over years of the study
    wsi.yrc[i] <- wsis[i]  # centered wsi in year i
    wsi.yr[i] <- wsis[i] + mean(wsis2)  # uncentered wsi in year i
    inds <- I(year == i)  # to pull off the right deer observations for the particular year
    ztemp <- zs[, deer.id[inds]]  # deer followed in year i (n.its x no deer in year i matrix)
    thetas <- jagsfit.mig$BU$sims.list$theta[, i]  # theta[0,t] for year i

    # Proportion of the study cohort in year t that is estimated to be an
    # obligate migrator (z=1) pi~.1 (eq. 5)
    pi1.tilde <- apply(ztemp, 1, mean)
    pi1.tilde.d[i, ] <- c(mean(pi1.tilde), quantile(pi1.tilde, probs = c(0.05,
        0.95)))

    # Proportion of the uncollared population that is estimated to be an
```

```
    # obligate migrator, pi.1
    pi1.d[i, ] <- c(mean(jagsfit.mig$BU$sims.list$pi.1[, i]), quantile(jagsfit.mig$BU$sims.list$pi.1[,
        i], probs = c(0.05, 0.95)))

    # To quantify the potential sample selection bias, take the difference
    # between pi~_1t and pi_1t
    diff.temp <- pi1.tilde - jagsfit.mig$BU$sims.list$pi.1[, i]
    diffpop.samp[i, ] <- c(mean(diff.temp), quantile(diff.temp, probs = c(0.05,
        0.95)))

    # ThetaP = marginal probability of migrating for the uncollared deer in the
    # population
    Thetap.temp <- jagsfit.mig$BU$sims.list$pi.1[, i] + (1 - jagsfit.mig$BU$sims.list$pi.1[,
        i]) * thetas
    Thetap[i, ] <- c(mean(Thetap.temp), quantile(Thetap.temp, probs = c(0.05,
        0.95)))

    # Lastly, we can calculate how many of the deer actually migrated in that
    # year The empirical proportion is stored in p_mig
    p_mig[i] <- mean(y[inds])
}
```

Code used to produce output in Figure 2A in the paper

```
# Calculate thetat|et=0 = prob(migrate | conditional migrator, et=0), these
# values will be stored in pconds.pi (point estimate & CI).
nvals <- 30
wsi.yr <- wsi.yrc <- matrix(NA, nvals, 1)
wsivals <- seq(40, 195, length = nvals)
pconds.ci <- matrix(NA, nvals, 3)
for (i in 1:nvals) {
    wsi.yrc[i] <- wsivals[i] - mean(wsis2)
    wsi.yr[i] <- wsivals[i]

    # thetat|et=0, probability a conditional migrator migrates (given et = 0,
    # i.e., a 'typical winter with WSI = x')
    pconds.temp <- plogis(jagsfit.mig$BU$sims.list$bo + jagsfit.mig$BU$sims.list$b1 *
        wsi.yrc[i])
    pconds.ci[i, 1] <- mean(pconds.temp)
    pconds.ci[i, 2:3] <- quantile(pconds.temp, probs = c(0.05, 0.95))
}
```

Figure 2 in the paper

```
if (spin == 0) {
    postscript("./output/fig1_ns.eps", horizontal = FALSE, onefile = FALSE,
        paper = "special", height = 6, width = 12)
}
```

```
## Error: comparison (1) is possible only for atomic and list types
```

```
par(mfrow = c(1, 2), cex.lab = 1.6, cex.axis = 1.6, cex.main = 1.6, oma = c(2,
    1, 1, 2), mar = c(4.1, 5.1, 4.1, 2.1), bty = "L", tcl = 0.5)

# PANEL A
plot(wsi.yr, pconds.ci[, 1], xlab = "Winter severity index (WSI)", ylab = "Proportion Migrating",
    ylim = c(0, 1), type = "l", col = gray(0.25), lwd = 3)
lines(wsi.yr, pconds.ci[, 2], lty = 2, col = gray(0.25), lwd = 3)
lines(wsi.yr, pconds.ci[, 3], lty = 2, col = gray(0.25), lwd = 3)
mtext(side = 3, line = 1, cex = 1.4, "A) ", adj = 0)
legend(125, 0.2, expression(paste(theta[list(0, t)], "|", epsilon[t] == 0)),
    lty = 1, col = c(gray(0.25), gray(0.001)), lwd = c(3, 1), bty = "n", cex = 1.4,
    adj = 0)
points(wsis2, p_mig, pch = 16, cex = 1.4)  # black points
points(wsis2, Thetap[, 1], pch = 16, col = "darkgray")
plotCI(wsis2, Thetap[, 1], li = Thetap[, 2], ui = Thetap[, 3], col = "darkgray",
    add = T, pch = 1, gap = 0.2, lwd = 1, cex = 1.3, lty = 2)

# PANEL B
```

```
yr <- 1991:2005
plot(yr, Thetap[, 1], type = "b", lty = 1, ylim = c(0, 1), xlab = "Year", ylab = "Proportion Migrating",
    xaxt = "n")
points(yr, Thetap[, 1], pch = 16, col = "darkgray")
lines(yr, Thetap[, 2], lty = 2)
lines(yr, Thetap[, 3], lty = 2)
points(yr, p_mig, pch = 16, cex = 1.4)  # Proportion in the sample
mtext(side = 3, line = 1, cex = 1.4, "B) ", adj = 0)
axis(side = 1, at = seq(1992, 2004, 4), labels = c("'92-'93", "'96-'97", "'00-'01",
    "'04-05'"))
```

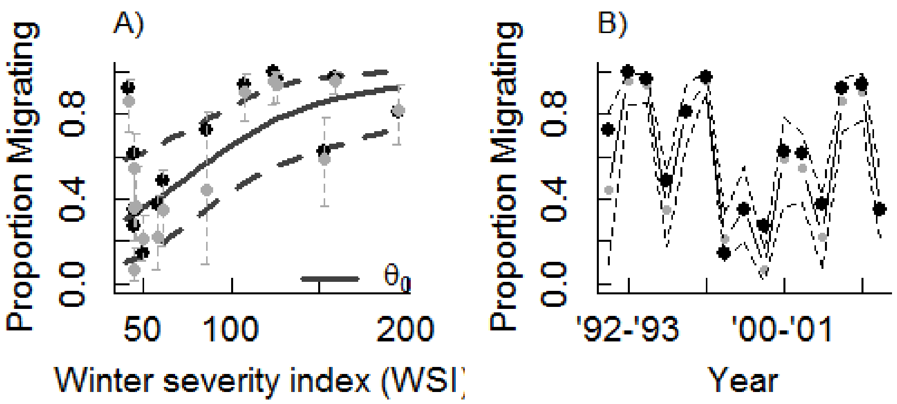


```
if (spin == 0) {
    dev.off()
}
```

```
## Error: comparison (1) is possible only for atomic and list types
```

Figure 3 in the paper

```
if (spin == 0) {
    postscript("./output/fig2_ns.eps", horizontal = FALSE, onefile = FALSE,
        paper = "special", height = 6, width = 18)
}
```

```
## Error: comparison (1) is possible only for atomic and list types
```

```
par(mfrow = c(1, 3), cex.lab = 1.8, cex.axis = 1.8, cex.main = 1.6, oma = c(2,
    1, 1, 3), mar = c(4.1, 5.1, 4.1, 2.1), bty = "L", tcl = 0.5)

# Panel A: pi.es versus time Panel B: pi.tild.es versus time Panel C:
# Selection bias versus time

# Panel A:
plotCI(yr, pi1.d[, 1], li = pi1.d[, 2], ui = pi1.d[, 3], xaxt = "n", xlab = "Year",
    ylab = expression(paste("Proportion of obligates in the population,   ",
        hat(pi[list(1, t)]))), ylim = c(0, 0.65), gap = 0.2, lty = 2, pch = 16,
    cex = 1.4)
axis(side = 1, at = c(1993, 1998, 2003), labels = c("'93-'94", "'98-'99", "'03-'04"))
mtext(side = 3, line = 1, cex = 1.4, "A) ", adj = 0)

# Panel B:
plotCI(yr, pi1.tilde.d[, 1], li = pi1.tilde.d[, 2], ui = pi1.tilde.d[, 3], xlab = "Year",
    ylab = expression(paste("Proportion of obligates in the study cohort,  ",
        tilde(pi[list(1, t)]))), ylim = c(0, 0.65), xaxt = "n", type = "l",
    gap = 0.2, lty = 2, pch = 16, cex = 1.4)
axis(side = 1, at = c(1993, 1998, 2003), labels = c("'93-'94", "'98-'99", "'03-'04"))
mtext(side = 3, line = 1, cex = 1.4, "B) ", adj = 0)
```

```
# Panel C:
plotCI(yr, diffpop.samp[, 1], li = diffpop.samp[, 2], ui = diffpop.samp[, 3],
    xaxt = "n", xlab = "Year", type = "l", ylab = expression(paste("Selection bias ,  ",
        tilde(pi[list(1, t)]) - hat(pi[list(1, t)]))), gap = 0.2, lty = 2, pch = 16,
    cex = 1.4)
axis(side = 1, at = c(1993, 1998, 2003), labels = c("'93-'94", "'98-'99", "'03-'04"))
par(new = T)
plot(yr, wsis2, ylim = c(-100, 200), new = T, col = "darkgrey", lwd = 2, yaxt = "n",
    xlab = "", ylab = "", type = "l", xaxt = "n")
axis(sid = 4, at = seq(50, 200, 50))
mtext(side = 4, line = 3, "Winter severity index (WSI)", cex = 1.8)
mtext(side = 3, line = 1, cex = 1.4, "C) ", adj = 0)
```

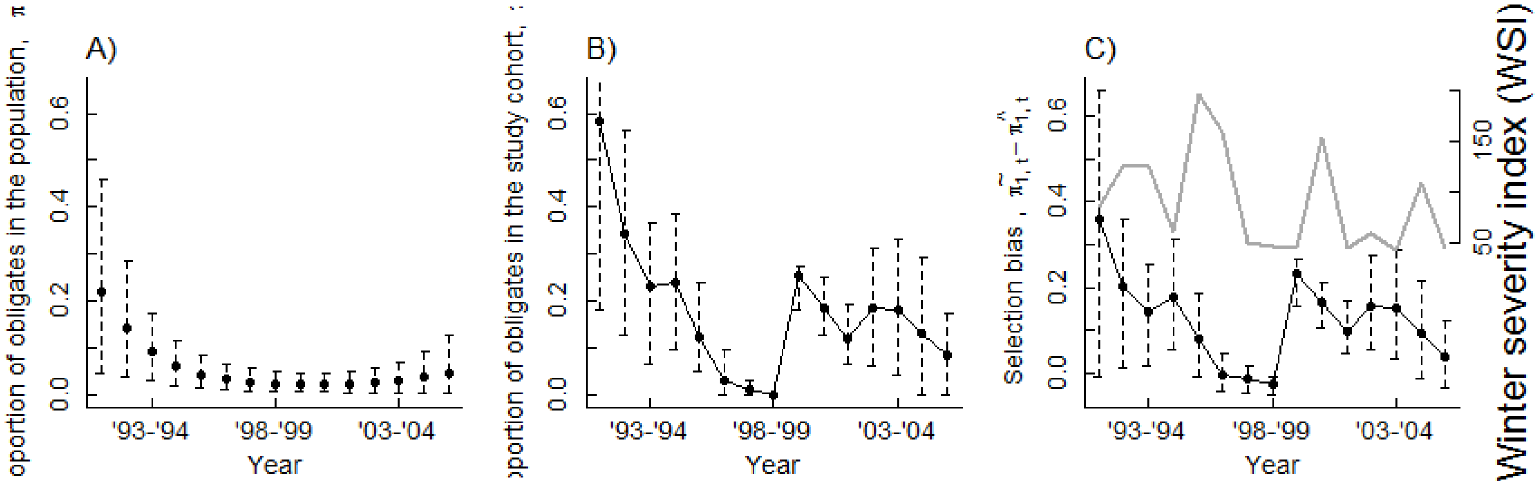


```
if (spin == 0) {
    dev.off()
}
```

```
## Error: comparison (1) is possible only for atomic and list types
```

Figure 4 in the paper Now, chart showing individual follow-up, with year of capture, and E[z=1] for each deer, along with wsi

```
E.z <- apply(jagsfit.mig$BU$sims.list$z, 2, mean)
```

Plot, E[z_i] as a function of WSI in year of capture

```
if (spin == 0) {
    postscript("./output/Ezplot.eps", horizontal = FALSE, onefile = FALSE, paper = "special",
        height = 6, width = 6)
}
```

```
## Error: comparison (1) is possible only for atomic and list types
```

```
par(mfrow = c(1, 1), cex.lab = 1.8, cex.axis = 1.8, cex.main = 1.6, oma = c(2,
    1, 1, 3), mar = c(4.1, 5.1, 4.1, 2.1), bty = "L", tcl = 0.5)
sunflowerplot(xyTable(wsis2[capyears], E.z), xlab = "WSI in year of capture",
    ylab = expression(E(z[i])), pch = 16)
```

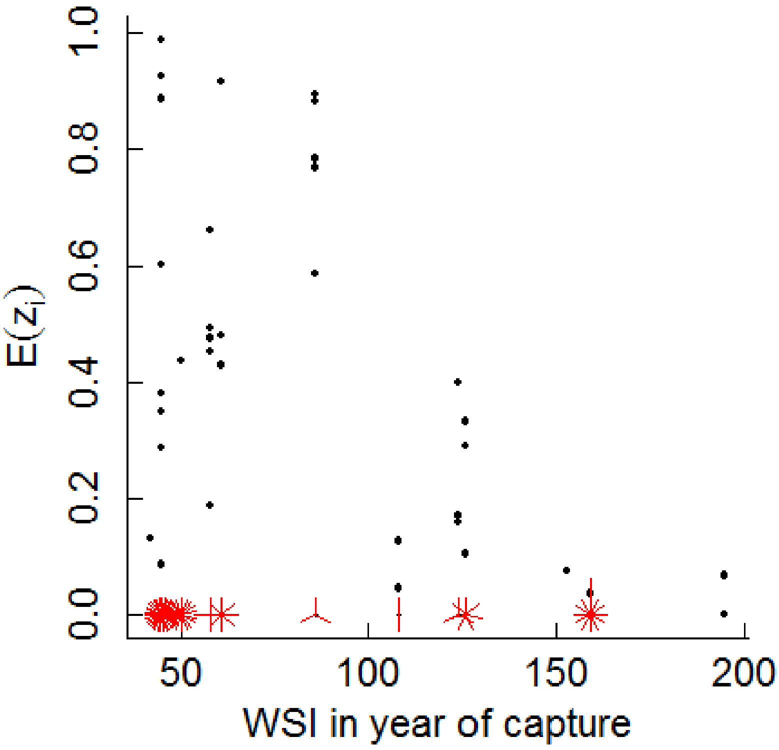


```
if (spin == 0) {
    dev.off()
}
```

```
## Error: comparison (1) is possible only for atomic and list types
```
